# Supplementary material for: A parsimonious approach for spatial transmission and heterogeneity in the COVID-19 propagation
Source: R Soc Open Sci. 2020 Dec 15;7(12):201382. doi: 10.1098/rsos.201382 (PMC7813252; doi:10.1098/rsos.201382)
Supplement: Supplementary Figures [file rsos201382supp1.pdf]

A parsimonious model for spatial transmission and heterogeneity in the COVID-19 propagation
SI 1. Supplementary figures

Lionel Roques, Olivier Bonnefon, Virgile Baudrot, Samuel Soubeyrand, Henri Berestycki

- The mean temperatures during the observation period are presented in Fig. S1, together with a scatter plot of the mean temperature vs immunity rate.

  (source: https://www.data.gouv.fr/fr/datasets/temperature-quotidienne-departementale-depuis-janvier-2018/).

- In Fig. S2, we describe the timeline of the spatio-temporal dynamics of the immunity rate during the observation period.

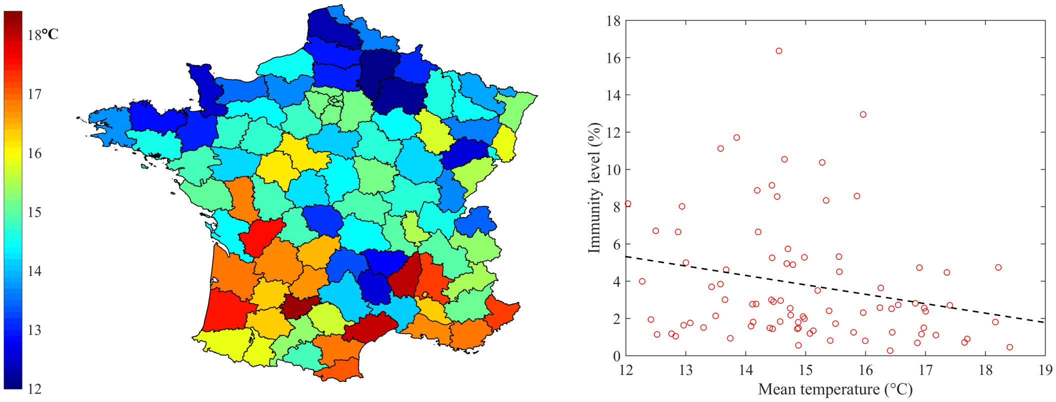

Figure S1: (a) Mean temperature in each county over the period ranging from 30 March 2020 to 11 June 2020. (b) Immunity rate vs mean temperature: we observe a negative correlation between the mean temperature and the immunity rate (Pearson correlation coefficient: -0.24).

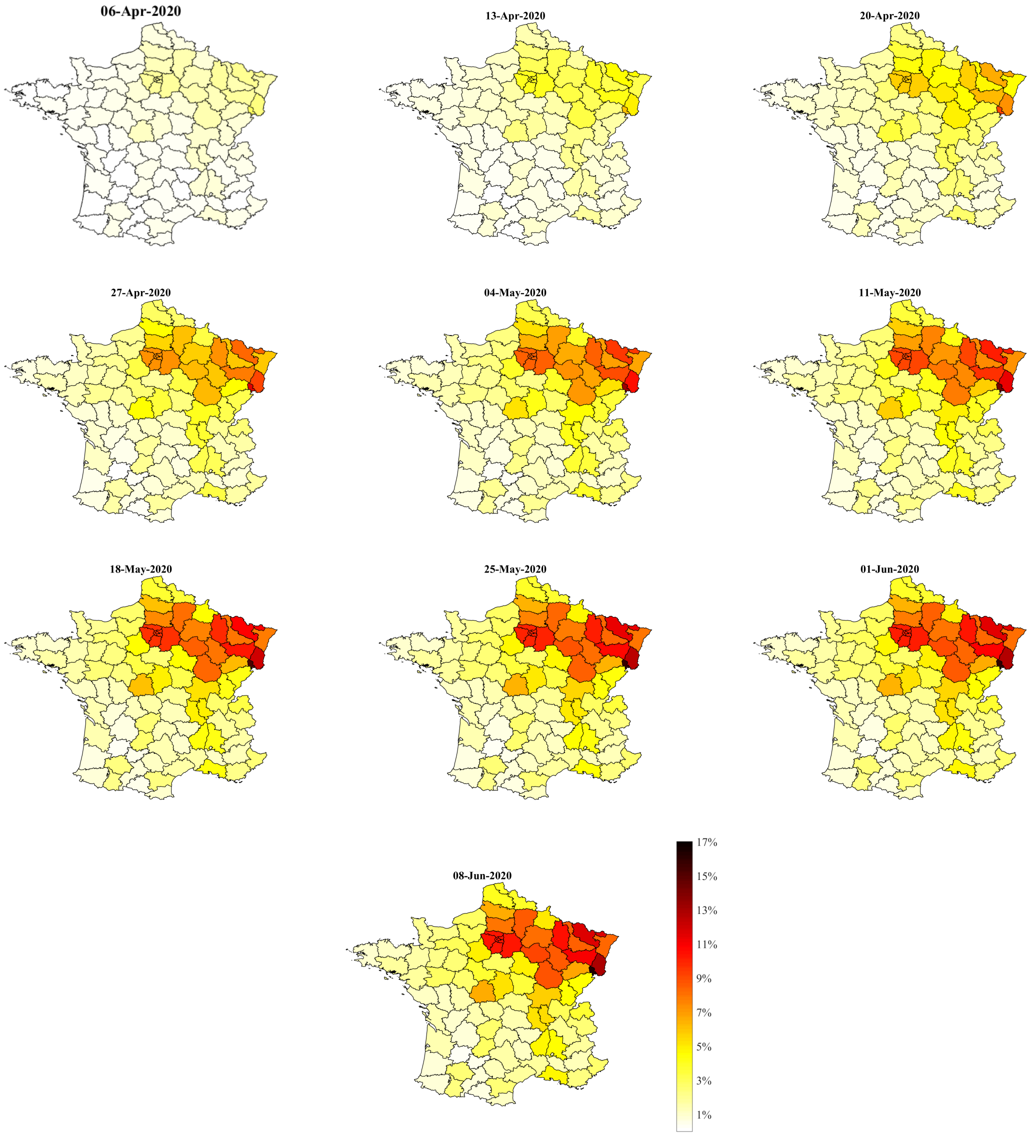

Figure S2: Dynamics of the immunity rate given by model $\mathcal{M}_3$, from 6 April to 8 June.
