# Supplementary material for: A parsimonious approach for spatial transmission and heterogeneity in the COVID-19 propagation
Source: R Soc Open Sci. 2020 Dec 15;7(12):201382. doi: 10.1098/rsos.201382 (PMC7813252; doi:10.1098/rsos.201382)
Supplement: Auxiliary models [file rsos201382supp2.pdf]

A parsimonious model for spatial transmission and heterogeneity in the COVID-19 propagation
SI 2. Auxiliary models

Lionel Roques, Olivier Bonnefon, Virgile Baudrot, Samuel Soubeyrand, Henri Berestycki

At each date t, the time-dependent parameters $\alpha(t)$ in $\mathcal{M}_0$ and $\mathcal{M}_1$ and $\alpha_k(t)$ in $\mathcal{M}_2$ are defined as the maximum likelihood estimators (MLEs) in the window $(t-\tau/2, t+\tau/2)$ of the following auxiliary models:

$$\begin{cases} \tilde{S}'(s) = -\dfrac{\tilde{\alpha}}{N}\,\tilde{S}\,\tilde{I}, \\ \tilde{I}'(s) = \dfrac{\tilde{\alpha}}{N}\,\tilde{S}\,\tilde{I} - (\beta+\gamma)\,\tilde{I}, \\ \tilde{R}'(s) = \beta\,\tilde{I}, \\ \tilde{D}'(s) = \gamma\,\tilde{I}, \end{cases} \quad \text{for } s \in (t-\tau/2, t+\tau/2), \qquad (\tilde{\mathcal{M}}_{0,t})$$

and

$$\begin{cases} \tilde{S}_k'(s) = -\dfrac{\tilde{\alpha}}{N_k}\,\tilde{S}_k\,\tilde{I}_k, \\ \tilde{I}_k'(s) = \dfrac{\tilde{\alpha}}{N_k}\,\tilde{S}_k\,\tilde{I}_k - (\beta+\gamma)\,\tilde{I}_k, \\ \tilde{R}_k'(s) = \beta\,\tilde{I}_k, \\ \tilde{D}_k'(s) = \gamma\,\tilde{I}_k, \end{cases} \quad \text{for } s \in (t-\tau/2, t+\tau/2), \qquad (\tilde{\mathcal{M}}_{1,t})$$

and

$$\begin{cases} \tilde{S}_k'(s) = -\dfrac{\tilde{\alpha}_k}{N_k}\,\tilde{S}_k\,\tilde{I}_k, \\ \tilde{I}_k'(s) = \dfrac{\tilde{\alpha}_k}{N_k}\,\tilde{S}_k\,\tilde{I}_k - (\beta+\gamma)\,\tilde{I}_k, \\ \tilde{R}_k'(s) = \beta\,\tilde{I}_k, \\ \tilde{D}_k'(s) = \gamma\,\tilde{I}_k, \end{cases} \quad \text{for } s \in (t-\tau/2, t+\tau/2). \qquad (\tilde{\mathcal{M}}_{2,t})$$

The initial condition in these models is computed iteratively from the solutions of $\mathcal{M}_0$, $\mathcal{M}_1$ and $\mathcal{M}_2$, respectively, over the period $[t_i, t-\tau/2]$.
